# A synthetic dataset of Danish residential electricity prosumers

**DOI:** 10.1038/s41597-023-02271-3

**Published:** 2023-06-08

**Authors:** Rui Yuan, S. Ali Pourmousavi, Wen L. Soong, Andrew J. Black, Jon A. R. Liisberg, Julian Lemos-Vinasco

**Affiliations:** 1 School of Electrical and Mechanical Engineering, The University of Adelaide, Adelaide, Australia; 2 School of Computer and Mathematical Sciences, The University of Adelaide, Adelaide, Australia; 3 Watts A/S, Køge, Denmark

**Keywords:** Energy supply and demand, Energy management

## Abstract

Conventional residential electricity consumers are becoming prosumers who not only consume electricity but also produce it. This shift is expected to occur over the next few decades at a large scale, and it presents numerous uncertainties and risks for the operation, planning, investment, and viable business models of the electricity grid. To prepare for this shift, researchers, utilities, policymakers, and emerging businesses require a comprehensive understanding of future prosumers’ electricity consumption. Unfortunately, there is a limited amount of data available due to privacy concerns and the slow adoption of new technologies such as battery electric vehicles and home automation. To address this issue, this paper introduces a synthetic dataset containing five types of residential prosumers’ imported and exported electricity data. The dataset was developed using real traditional consumers’ data from Denmark, PV generation data from the global solar energy estimator (GSEE) model, electric vehicle (EV) charging data generated using emobpy package, a residential energy storage system (ESS) operator and a generative adversarial network (GAN) based model to produce synthetic data. The quality of the dataset was assessed and validated through qualitative inspection and three methods: empirical statistics, metrics based on information theory, and evaluation metrics based on machine learning techniques.

## Background & Summary

With the increasing penetration of renewable energy sources (RES), electric vehicles (EVs) and energy storage systems (ESS) in modern households, conventional consumers are changing into prosumers, making the power systems increasingly dynamic and bidirectional. In 2022, RESs continued their rapid growth, accounting for 13% of global power generation, showing a 17% increase compared to 2021^1^. The International Energy Agency (IEA) outlook, published in 2021, predicted 56% of global electricity generation to come from renewables by 2050, where solar is projected to be the primary renewable resource taking up to 43% of the total RES share^2^. Global electricity consumption will also increase due to space heating and transportation electrification. Amongst all the electricity usage, domestic EVs are believed to be the major contributor to emissions reduction, expected to represent 70% of total passenger vehicles by 2050, whilst battery electric vehicles (BEV) will account for 56% of all vehicle sales^3^.

Based on this projection, it is imperative for grid operators, policymakers, utilities and other stakeholders to understand the dynamics of residential electricity consumption in the future. However, there are several barriers to this, mainly regarding high-quality data availability. First, large-scale individual electricity consumption data is unavailable to practitioners and researchers due to consumers’ privacy concerns. In countries with widespread smart meter rollouts, interval consumption data is available only to consumers, system operators and retailers for billing. However, in all cases, the types of users based on their behind-the-meter (BTM) equipment, e.g., EV, stationary batteries or solar PV systems, are unknown. Second, the existing electricity prosumers’ type is quasi-dynamic and changes over time with no mechanism to update the categorisations of prosumers. For example, a solar PV malfunction can make a solar user temporarily a non-solar user or the unavailability of an EV can temporarily change the user’s type. Dynamic knowledge of the prosumer type (e.g., on an hourly or daily basis) could be crucial for system operators, aggregators and retailers to better estimate the demand behaviour in hours to days ahead for planning and operation. In this regard, a large-scale labelled dataset of different types of prosumers’ electricity consumption facilitates the modernisation of power grids^4^.

Existing public datasets fall into two major categories: (1) harvested data from living labs^5–7^ and (2) simulation studies^8,9^. Some living labs worldwide gather appliance-level interval data with smart meters and other smart devices^6,7,10,11^. These can provide high-resolution data but only for a limited number of prosumers. Due to privacy concerns or contractual obligations, some of them cannot share data publicly. Of simulation studies, some researchers have built either physics-based or data-driven models for simulating individual household electricity usage^8,9,12^. The physics-based models require physical parameters of the buildings, such as thermal capacitance, thermal resistance, indoor temperatures, etc., which are difficult to obtain and maintain in practice. Moreover, the physics-based models exacerbate privacy concerns because the more it knows about a prosumer, the easier it is to identify the household. Compared to the physics-based models, the data-driven models rely only on historical data of consumers/prosumers. The main issue is that residential BTM technologies with appropriate automation have not been adopted at a large scale yet, particularly for stationary batteries and BEVs. Therefore, the data-driven models do not have enough interval data to synthesise a wide variety of different types of prosumers’ time series.

To solve the data availability issue, we first build a dataset based on real-world consumers’ data as benchmark users and aggregate it with three different RES interval data considering other information from Denmark. The three considered RESs are: automated energy storage systems (ESS), rooftop solar PV systems and BEVs, as it is expected for BEVs to dominate the future vehicle market^3^. This way, we create five prototypes of prosumers and one prototype of consumers for the sake of completeness. To tackle the privacy concern of using real-world consumers’ data, we reformat the data in a daily manner and apply conditional tabular generative adversarial network (CTGAN)-based data synthesizers to generate synthetic data for each prototype. This procedure can protect the privacy of real-world consumers for three reasons. First, we used real consumers’ electricity data to produce different types of prosumers’ electricity profiles, which means that their true consumption is concealed by mixing it with the RES time series. Secondly, the data generator is a black-box method that cannot be reverse-engineered and is hard to disaggregate. Additionally, the end user’s lifestyle and occupancy are non-existent in the dataset because the dataset contains only daily profiles under certain seasons and temperatures; hence, there is no connection between two consecutive days. Overall, we created a synthetic dataset of 600,000 days of imported energy from the grid and exported energy to the grid. The devised algorithm produces six types of electricity users’ consumption profiles considering two types of days (weekday and other days, which includes public holidays and weekends), four seasons, and ambient temperature. Notably, we target Danish residential prosumers because our industrial partner, Watts A/S, is from Denmark and provided traditional consumers’ hourly usage data for our project^13^. Nevertheless, the proposed data synthesizer is generic and can be used to synthesise data for other regions and countries contingent on data and required information availability.

Several factors make this study and dataset significant. Firstly, the dataset contains hourly imported (from the grid) and exported (to the grid) electricity usage of individual residential users labelled by BTM equipment, type of day, season, and daily temperature. To the best of our knowledge, such a dataset is not currently available to the public for research and development^7,14^. Also, the dataset can be used in different applications, e.g., system planning, market analysis and business model development, BTM flexibility modelling, community energy hubs design, microgrid and local market design, and electrification assessment and its impact on greenhouse gas emissions in the prosumers’ era^15,16^. Secondly, the dataset’s quality is validated in four ways, i.e., qualitative inspection, empirical statistics, Machine Learning (ML) based evaluation metrics, and information theory. Finally, the synthetic dataset sidesteps the privacy concerns because of the reasons discussed above.

## Methods

This section describes the methodology of generating the proposed synthetic dataset, including an overall workflow, residential BEV consumption modelling, residential PV generation modelling, and automated ESS modelling for synthesising the data. Finally, we introduce the CTGAN used for synthetic data generation.

### Overview

**Fig. 1:**
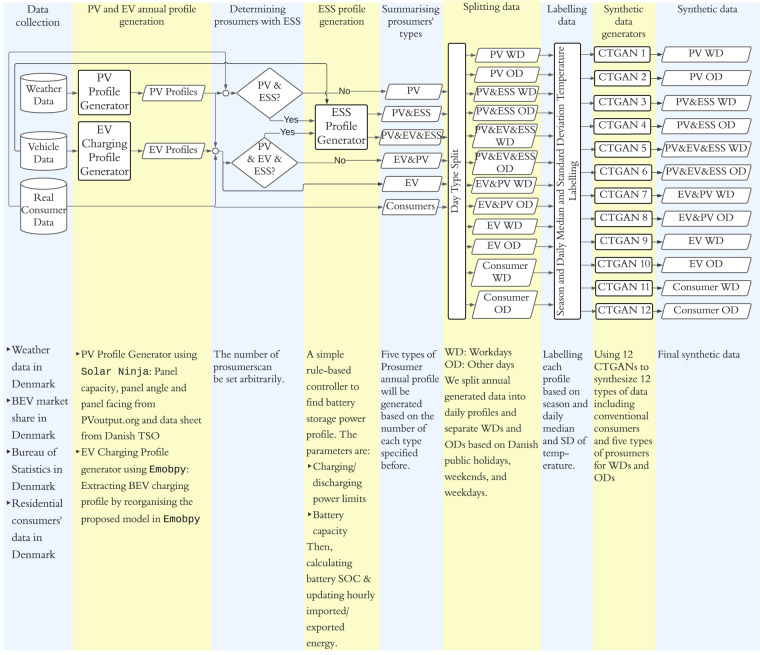
Data generation process break down.

The block diagram in Fig. 1 shows the workflow of our methodology. In general, eight phases are involved in obtaining the final synthetic dataset. These phases include data collection, generation of PV and EV annual profiles, determining prosumers with ESS, generation of ESS profiles, summarising prosumers’ types, data splitting, data labelling, and synthetic data generation. In the data collection stage, we utilise energy data from 2,000 real Danish consumers, including imported and exported energy data in hourly resolution for 2019, provided by our project industry partner. These profiles serve as the baseload. These are Danish residential households living in the same neighbourhood under the same weather conditions. The raw data was collected from the DataHub^17^ of the Danish transmission system operator (TSO) EnergiNet, with consumers’ consent following the industry partner’s privacy policies^18^, General Data Protection Regulation (GDPR)^19^, and Danish Data Protection Act^20^. The weather data is collected from OpenWeather for the specific area^21^ and down-sampled to match the energy data resolution, i.e., hourly resolution. While the BEV adoption rate has exponentially increased over the last few years^22^, there are insufficient BEV owners willing to share their data to help build a credible dataset. Additionally, most current BEV owners use slow chargers at home, and their BEV charging consumption is not recorded separately. Therefore, we need a sophisticated BEV charging data model to generate data for Danish EV owners under different scenarios. We use a trustworthy, validated tool^23^ with many features and functionalities to simulate the EVs’ charging demand in Denmark’s residential sector in detail. To incorporate Danish driving habits, we collected Danish mobility statistics on the number of trips per day, distance and duration, BEVs specifications such as motor type, battery size, heat transfer, and other external factors such as charging station availability and power rating of the chargers from the Bureau of Statistics and BEV market share in Denmark^24,25^. More details about BEV charging data are presented in Section ‘EV Profile Generator’.

We had the same data availability problem with residential PV generation data. By the end of 2019, only 13% of Danish households owned rooftop PV systems^26^. Also, PV generation is not separately metered; only exported energy data is available. Therefore, we use a PV generation model taking into account local weather information and systematic biases on the Meteosat-based satellite dataset. The process of synthesizing PV generation data is explained in detail in Section ‘PV Profile Generator’. Having EV and PV profiles in hand, a further consideration is whether the prosumers have a stationary battery at home, which is done in the stage of determining prosumers with ESS. Here, we arbitrarily selected 300 prosumers as ESS users due to a lack of data about the current status of residential energy storage in Denmark. Nevertheless, one can assume different penetration levels to see the impact on the prosumers’ imported/exported energy profile. For consumers with a stationary battery at their premises, a rule-based automation system is developed to produce the battery’s charging/discharging profiles according to internal consumption, PV generation and BEV consumption (if any). The rule-based controller for residential ESS operation is the most common approach in the industry nowadays^27^. We explain the ESS data generation in Section ‘ESS Profile Generator’.

With the three models and real consumers’ data, we built a seed dataset including five types of prosumers with different combinations of BTM equipment. The dataset is then split into daily profiles with two types of days, i.e., weekdays and other days, including holidays and weekends. As shown in the diagram of Fig. 1, we label the generated data according to the two types of days (workday or other), the median temperature of the day, the standard deviation of the daily temperature, and four seasons to generate a synthetic dataset with 12 CTGANs based on their user types. Further details are provided in ‘Synthetic data generative model’.

The electricity usage profiles vary significantly from region to region for many socio-economic, cultural, and technical reasons. While we used the proposed framework to synthesise Danish residential prosumers’ and consumers’ data in this study, the proposed framework can be applied to synthesise prosumers’ and consumers’ data in any region by customising the input parameters.

### EV profile generator

The BEV charging activity is simulated with the emobpy^23^ in PYTHON. emobpy is an open-source tool that allows the generation of BEV charging profiles from empirical mobility statistics and physical properties of vehicles. It models individual BEVs’ driving mobility, electricity consumption, grid availability and the imported energy from the grid for a household using four sequential models. Specifically, the vehicle mobility model uses a sampling approach to generate plausible travelling routines for each day of the calculation period based on empirical probability distributions. The output of this model is a chronologically sorted list of trips, represented by edges connecting origin and destination locations with departure time, distance travelled, and trip duration. The electricity consumption model estimates a time series of driving electricity consumption of BEVs during driving. It formulates the power requirements for vehicle traction, heating and cooling by considering the vehicle mobility time series generated by the vehicle mobility model, vehicle type, speed, and terrain. The grid availability model takes into account the driving electricity consumption and the availability of charging infrastructure to determine the grid availability time series, which represents the percentage of time when charging is possible for BEVs in a given area. Lastly, the imported energy from the grid model generates a time series of grid electricity demand to charge BEVs based on the driving electricity consumption time series and grid availability generated by the previous models.

To repurpose the tool for our application, we integrated the four models introduced in emobpy into one model and customised settings to build a new model that takes the BEV physical properties and weather conditions as inputs and extracts the residential BEV charging profile as the output. The input parameters, shown in Fig. 1, are collected based on the BEV market sharing statistics in Denmark^25^ and employment data from Statistics Denmark^24^. Considering the total amount of data and excluding the failure cases, we generated 743 BEV users’ residential charging profiles for a year, including different employment statuses, i.e., full-time, part-time, and free-time BEV users and different BEV brands based on the above statistics. Hence, we produced 538 full-time users, 178 part-time users and 30 free-time users’ BEV charging profiles that will be used later to synthesise many more BEV users. For simplicity and because we do not involve hybrid EVs in the study, we labelled BEV users as EV users in the dataset hereafter. In addition, we do not consider vehicle-to-grid operation in this paper.

### PV profile generator

We used solar ninja to generate PV profiles. The tool uses the global solar energy estimator (GSEE) model to represent rooftop solar systems behaviour together with the global meteorological reanalyses and Meteosat-based CM-SAF SARAH satellite dataset to produce hourly PV generation profiles^28^. To be more specific, the tool uses mathematical modelling to estimate the power output of PV panels by calculating solar irradiance on the plane of the PV, as well as accounting for inverter and system losses caused by temperature-dependent panel efficiency curves. Hence, the model is deterministic and requires inputs of diffuse irradiance, direct irradiance, temperature, latitude, longitude, system loss, tilt, rated capacity of the panels, panel angle and panel orientation. The GSEE model has been validated across several European countries in various research studies, e.g.,^29–31^. To leverage the capabilities of this tool in our study, except for the weather and geographical parameters, other input parameters (e.g., PV capacity, losses and tilt) are obtained from the PVoutput platform^32^, which is a public sharing platform for residential PV generation data. Furthermore, we used data sheets from the Danish TSO to extract typical parameters, such as PV capacity, tilt and system loss, for small residential PV systems in Denmark^33^. With these inputs, representative models are built to synthesise PV generation data for further use in this study.

### ESS profile generator

Most of the research on energy storage technologies in Denmark falls into two types: centralised solutions and residential level storage, whereas the studies are generally from an aggregated level as users with ESS tend to be modelled as a group^34–37^. In our proposed dataset, we assume the ESS is owned by residential users and operates using a simple rule-based controller (a common practice in the industry called the naive operation method)^27^. The study shows the naive operation method has comparable performance to complicated stochastic optimisation models for most of the cases^27^. To simulate ESS operation, two parameters are required, namely charging capacity (maximum usable energy storage *S*_max_) and the charging/discharging power limit *P*_max_. These two parameters are generated using the probability distribution of different ESS brands based on their market share from our industry partner^13^ and ESS specifications in^38,39^. The rule-based battery controller operates as follows, assuming the State of Charge (SoC) at time *t* is *S*_*t*_:

1. When the net demand is positive, i.e., generation is larger than demand (*E*_g, *t*_>*E*_d, *t*_), the battery charging power, hence hourly energy, will be ${\rm{\min }}\left({E}_{{\rm{g}},t}-{E}_{{\rm{d}},t},{P}_{{\rm{\max }}},{S}_{{\rm{\max }}}-{S}_{t}\right)$, where imported energy is zero, and the exported energy will be:
  1 $$\begin{array}{c}{E}_{{\rm{g}},t}-{E}_{{\rm{d}},t}-{\rm{\min }}\left({E}_{{\rm{g}},t}-{E}_{{\rm{d}},t},{P}_{{\rm{\max }}},{S}_{{\rm{\max }}}-{S}_{t}\right)\\ =\,{\rm{\max }}\left(0,{E}_{{\rm{g}},t}-{E}_{{\rm{d}},t}-{P}_{{\rm{\max }}},{E}_{{\rm{g}},t}-{E}_{{\rm{d}},t}-{S}_{{\rm{\max }}}+{S}_{t}\right).\end{array}$$
2. When the net demand is negative, i.e., generation is lower than or equal to demand (*E*_g,*t*_ > *E*_d,*t*_), exported energy will be zero. Hence, the battery discharge power is equal to ${\rm{\min }}\left({E}_{{\rm{d}},t}-{E}_{{\rm{g}},t},{P}_{{\rm{\max }}},{S}_{t}\right)$, and the imported energy will be:

2 $$\begin{array}{c}{E}_{{\rm{d}},t}-{E}_{{\rm{g}},t}-{\rm{\min }}\left({E}_{{\rm{d}},t}-{E}_{{\rm{g}},t},{P}_{{\rm{\max }}},{S}_{t}\right)\\ =\,{\rm{\max }}\left(0,{E}_{{\rm{d}},t}-{E}_{{\rm{g}},t}-{P}_{{\rm{\max }}},{E}_{{\rm{d}},t}-{E}_{{\rm{g}},t}-{S}_{t}\right).\end{array}$$

Using the naive operation method described above, the battery will be charged when excess PV generation is available. The battery would be discharged to minimise imported energy from the grid when household electricity demand is higher than PV generation.

### Synthetic data generative model

With the EV, PV and ESS profile generators, we build a dataset including five types of prosumers and one type of consumer. To tackle the privacy concern discussed in ‘Background & Summary’, we split each user’s time series into separate days, aggregated these into daily profiles and then used them as inputs to generate a synthetic dataset. Other input parameters are the daily median and standard deviation of temperature as continuous variables, along with season being a categorical variable. To summarise, the parameters are as follows:

- Type of the days
  - Workdays (252 days): All weekdays excluding holidays.
  - Other days (113 days): Public holidays and weekends.
- Major BTM equipment
  - PV
  - PV & ESS
  - PV & EV
  - PV & EV & ESS
  - EV
  - Conventional consumers
- Temperature
  - Daily median temperature
  - Daily standard deviation of temperature
- Seasons (spring, summer, autumn and winter)

There are many techniques to synthesise time series, including copula-based models^40,41^, flow-based models^42^, diffusion models^43,44^, and GAN models^45–47^. Although diffusion models perform better in generating synthetic images, GAN-based models are preferred in synthesising time series because of their ability to generalise and produce a variety of high fidelity of data^48–50^. In this paper, we use the CTGAN model, which contains a conditional GAN and two techniques to generate synthetic data from tabulated real data. More specifically, the CTGAN applies a training-by-sampling technique for categorical columns and uses a variational Gaussian mixture model (VGM) instead of a GMM (Gaussian Mixture Model) for numerical columns to accurately model complicated distributions. In this study, we have 12 types of prosumers/consumers (based on the BTM equipment and type of day listed above); hence, 12 CTGANs, as shown in Fig. 1. Then, the 12 CTGAN models are trained based on each user type of data. With those 12 types of user models, we generate a balanced synthetic dataset. The user distribution ratio between the real and synthetic datasets is shown in Fig. 4. The hyperparameters of the CTGANs are identical in all 12 models and set as shown in Table 1.

**Table 1:** Hyperparameters for CTGAN.

Hyperparameter	Value
Epoch	300
Embedding dimension	128
Optimiser	Adam
Batch size	500
Learning rate for Generator	2e-4
Learning rate for Discriminator	2e-4

## Data Records

**Fig. 2:**
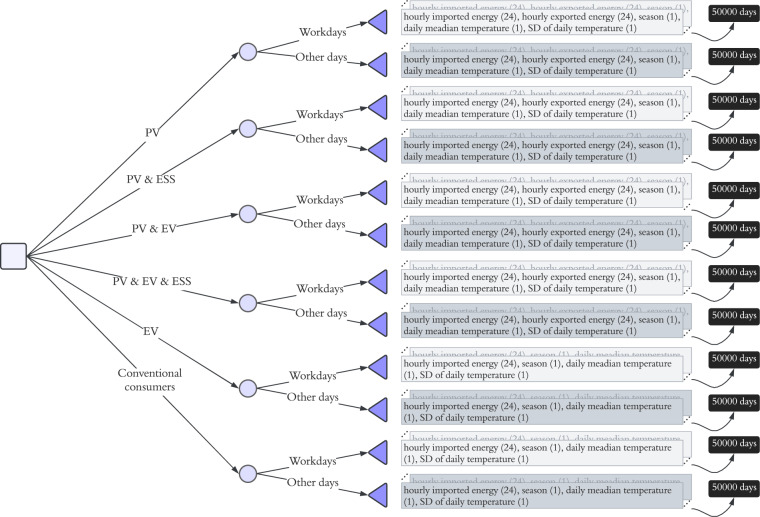
Structure of the proposed dataset.

Using the discussed framework in Fig. 1, the final synthetic dataset was generated. The dataset is made available to the public at Figshare^51^ in two formats, namely a pickle file with the same structure as in Fig. 2 for exclusive use in PYTHON, and an XLSX file for users who are not familiar with computational tools^51^. Specifically, the pickle file is a nested object containing six types of users by their major equipment, namely PV users, PV & ESS users, PV & EV users, PV & EV & ESS users, EV users and conventional consumers, respectively. Each type of user has two types of days, i.e., workdays and other days, which include imported and exported energy, daily average temperature, daily temperature standard deviation, and season. On the other hand, the XLSX file presents six types of users’ imported & exported energy under two types of days, each with its own formatted spreadsheet. Notably, there are 20 spreadsheets/tabs in total as EV users and conventional consumers do not have renewable generation, hence no exported energy. The columns of each spreadsheet are the 24 hourly timestamps in a day, i.e., 0–23, the median temperature, the standard deviation of the temperature, and the season of the day. In the online repository^51^, we also explained how to convert the XLSX worksheet to a CSV file for the convenience of users applying computational tools other than the ones in PYTHON.

**Fig. 3:**
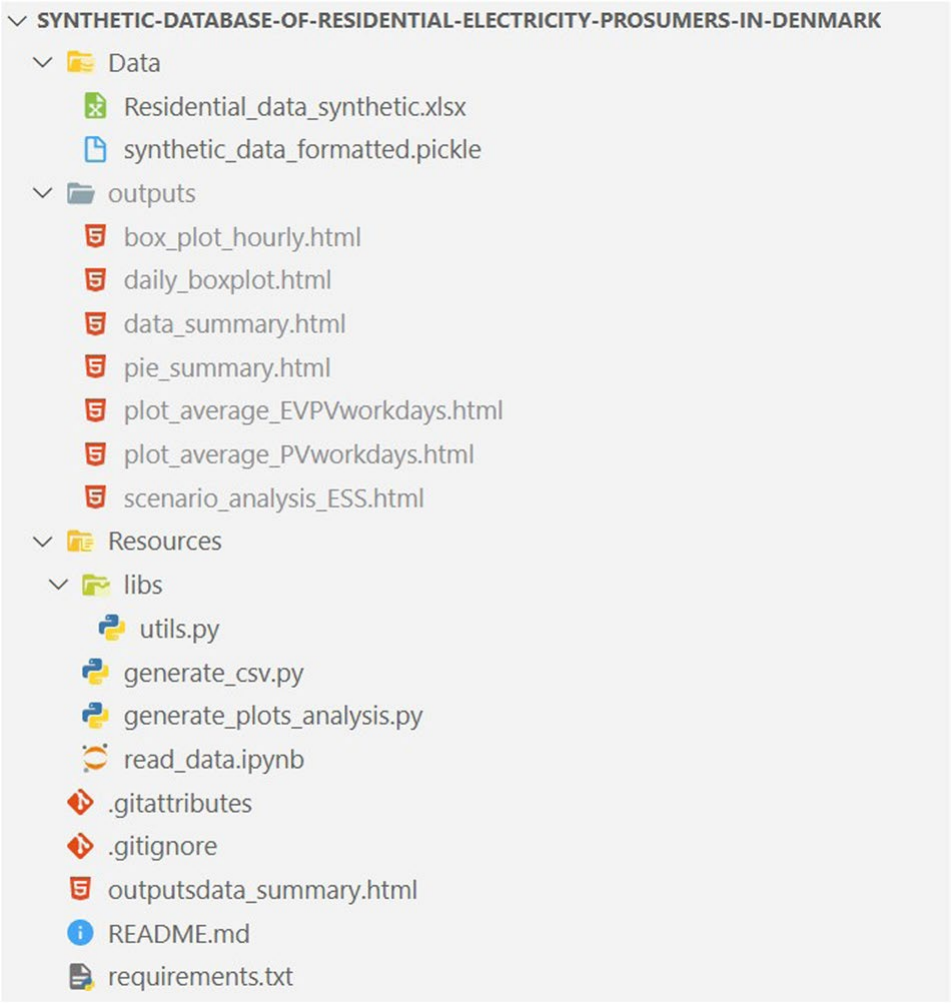
Dataset file structure.

The public repository contains the files as shown in Fig. 3, where the Data folder contains the proposed dataset in two formats, including pickle and XLSX^51^. The Resources folder contains codes in PYTHON for data conversion and data analysis. The outputs folder includes the generated visualised results from running the plot analysis code ‘generate_plots_analysis.py’ in the Resources folder. The requirements file outlines the dependencies used in this project^51^.

**Fig. 4:**
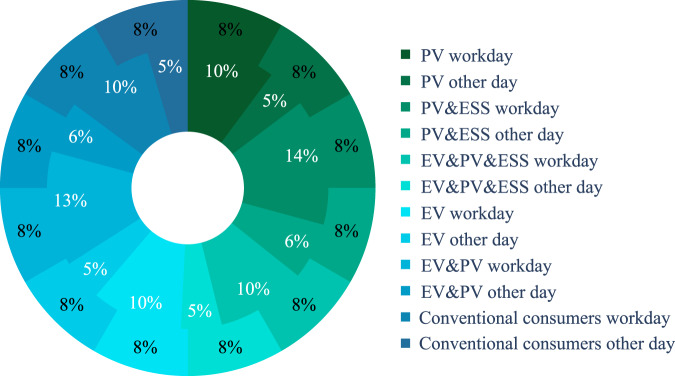
User type distributions (inner doughnut: real data, outer doughnut: synthetic data).

## Technical Validation

We validated the quality of the synthetic data using qualitative inspection and three numerical analyses: empirical statistics, metrics based on information theory, and ML-based evaluation metrics^52^. As discussed in the ‘Background & Summary’, a labelled large-scale real prosumers dataset does not exist. Therefore, we take the input seed dataset for the synthetic data generative model as the real dataset for validation purposes. We discuss each validation method respectively in the four subsections below.

### Qualitative inspection

**Fig. 5:**
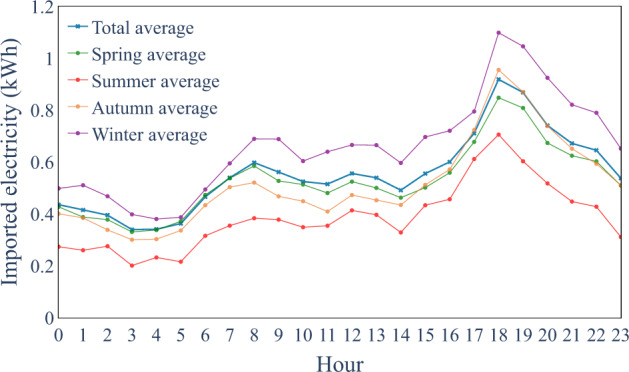
Comparison of seasonal demand profile for conventional consumers.

**Fig. 6:**
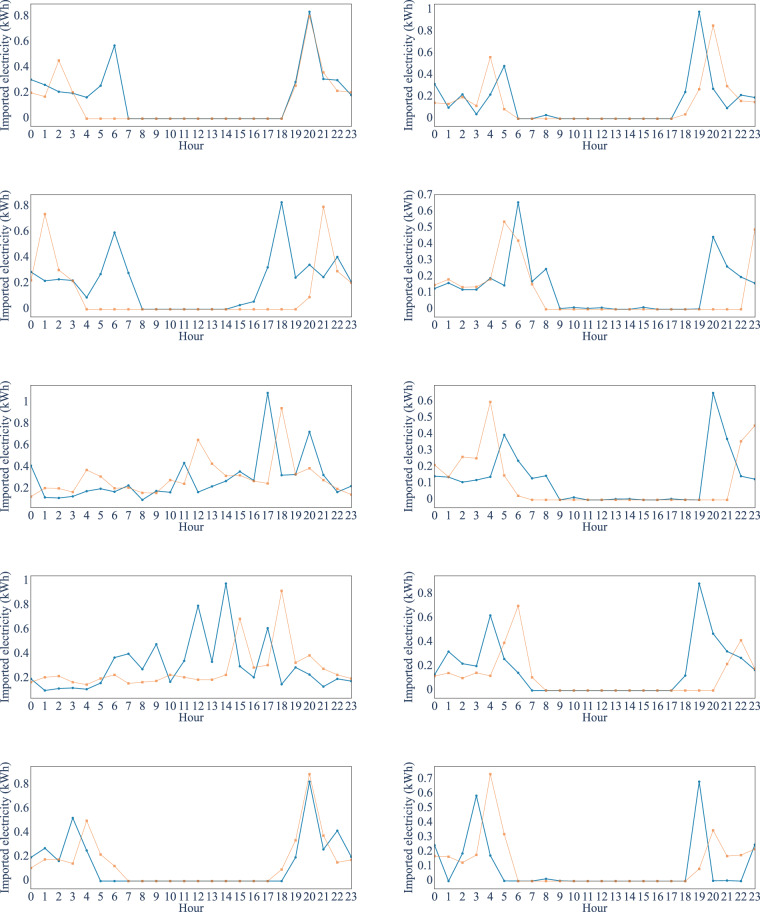
RM comparison for real and synthetic data (blue: synthetic data, orange: real data).

We compared the average seasonal consumption of the conventional consumers on weekdays in Fig. 5. This average profile is studied and compared to the real Danish residential electricity consumption characteristics on an aggregated level^11,53^. The general profile shape and the peak hour of imported electricity at 7 pm are similar. Besides the average consumer profiles, we compared the most frequent daily patterns for each prosumer type, called Refined Motifs (RM), between the real and synthetic datasets^4^. The results are shown in Fig. 6 for different types of prosumers and days. The RMs for synthetic data and real data share similar amplitude and trend, which indicates the synthetic dataset has similar shapes to the actual dataset^4^.

### Empirical statistics

**Fig. 7:**
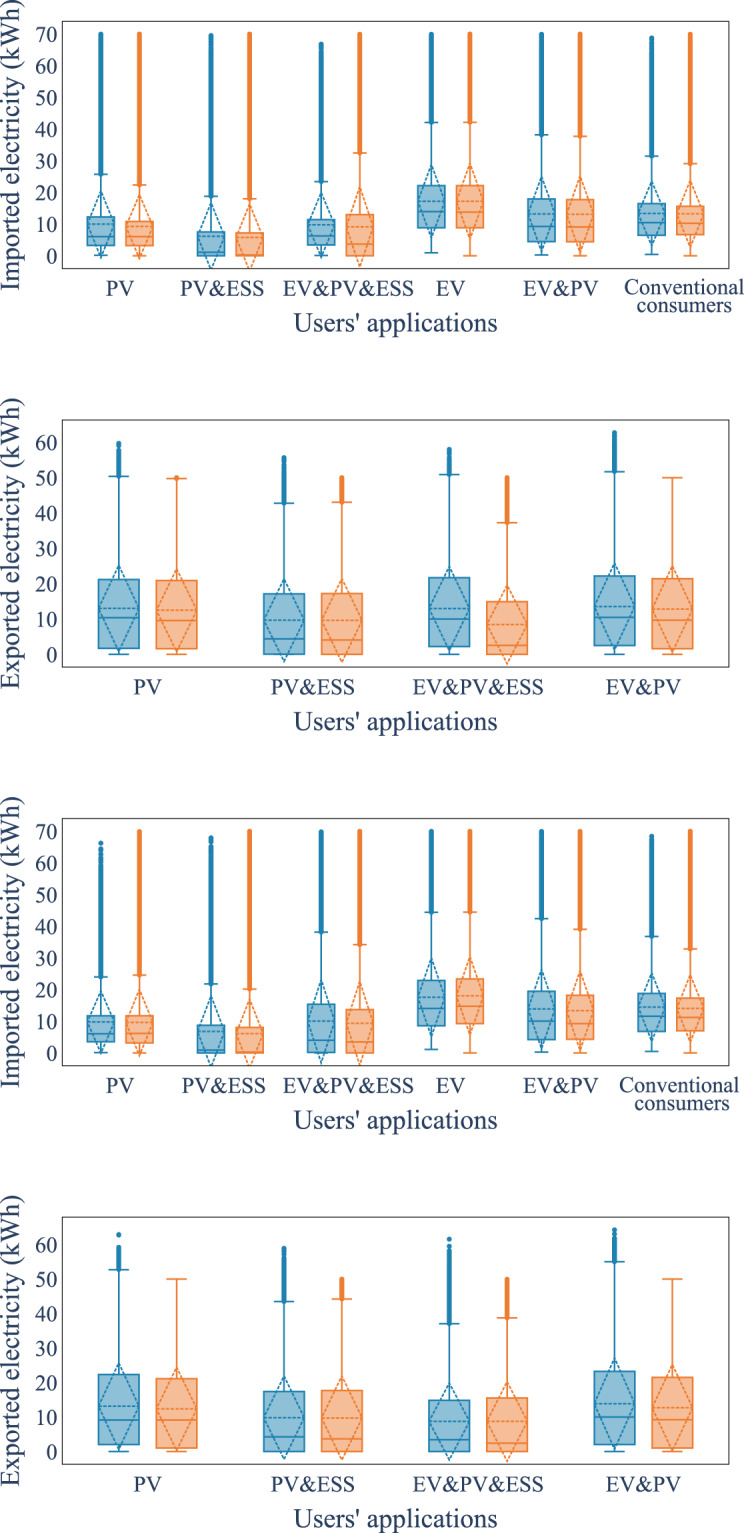
Daily statistics (blue: synthetic data, orange: real data).

**Fig. 8:**
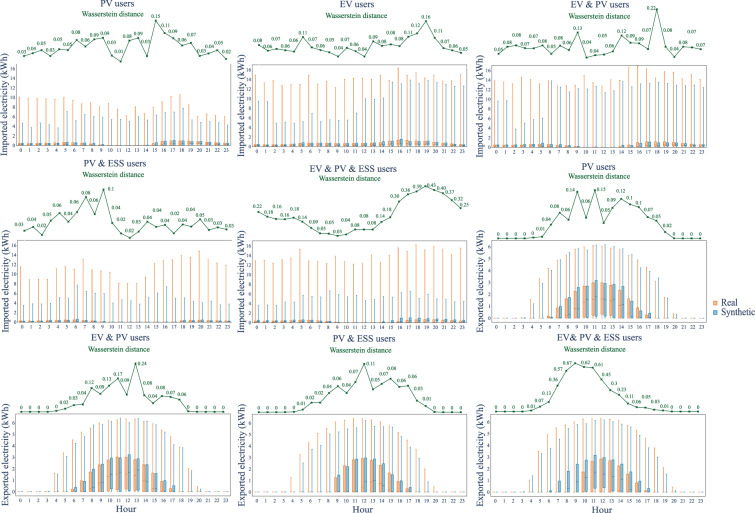
Hourly statistics on workdays. (green: Wasserstein distance between synthetic and real data. blue: box plot of synthetic data, orange: box plot of real data).

We firstly used box plots to visually compare the empirical statistics of the real and synthetic datasets, including the degree of dispersion (spread) and skewness of the two datasets, 1st and 3rd quartiles, interquartile range, mean, median, minimum, maximum, and outliers. The first comparison is made for the aggregated data, shown in Fig. 7 separately for different types of day and imported/exported energy. Overall, the synthetic data statistics follow the values of the real dataset. The workday imported energy dataset shows the highest errors for PV, EV and ESS users, while the other days’ statistics are almost identical. We also compared the hourly energy imported and exported box plots by hours for each type of user in the synthetic and real datasets, shown in Fig. 8, where the synthetic data follows the general trend in every figure. To quantify the difference between the real and synthetic data distributions, the Wasserstein distance, a metric of the distance between two probability distributions^54^, is computed for each interval. The lower Wasserstein distance values indicate greater similarity or overlap between the real data and synthetic data distributions. From the box plots in Fig. 8, it appears that the synthetic dataset has a lower maximum value than the real data for some user types, e.g., PV & EV & ESS users and PV& EV users. One reason could be the loss function in the CTGAN, evidence of lower-bound (ELBO) loss, which omits the abnormal data from the real dataset in the optimisation process. From the Wasserstein distance, PV & EV & ESS users exhibit the largest differences between the synthetic and real datasets among all types of users. This observation is further supported by the daily data box plots, which provide detailed information on the interquartile range differences. Specifically, the largest mismatches for PV & EV & ESS users tend to occur around 8–11 am for exported electricity and 7–8 pm for imported electricity. These time periods coincide with high stochasticity in the generation and demand data of prosumers due to the influence of PV generation, EV charging and ESS operation. Consequently, this discrepancy leads to higher differences in the aggregated level empirical statistics between the synthetic and real datasets.

### Information theory metrics

Permutation Entropy (PE) is a well-known time series information theory metric that quantifies the complexity of a dynamic system by capturing the order relations between the values of a time series and extracting a probability distribution of the ordinal patterns^52^. In an attempt to overcome some limitations, e.g., being incapable of differentiating between distinct patterns and insensitivity to patterns close to the noise floor, which makes it unsuitable for applications like power system data analysis^55^, the Weighted Permutation Entropy (WPE) was proposed as a measure with more robustness and stability by incorporating amplitude information^55,56^.

We used the WPE measure to compare the complexity of the synthetic dataset to the real dataset for each type of user. The WPE hyperparameters are set to the order of 6 and delay of *τ* = 1 based on the recommendations in^57,58^. A comparison between real and synthetic data is presented in Table 3. In ideal conditions, we expect both datasets to have similar complexity, i.e., WPE values. From the table, we can see that the synthetic dataset is more complex than the real data, as the WPE for the synthetic dataset is higher. However, the relative relationship between different types of users is consistent from real to synthetic datasets, where the synthetic dataset is always more complex despite the user type. To prove the robustness of this feature, we split the dataset into 50 time-series with one year’s worth of data for both real and synthetic datasets. Then, we calculated the WPE for each time series, shown in Fig. 10. As expected, the synthetic dataset always shows a higher complexity across different types of users, although the average WPE values are close between the real and synthetic datasets. This shows that the CTGAN generally overestimates the complexity of the real dataset. However, the user types with higher complexity in the synthesised dataset correspond to the same type in the real dataset, which means the models can successfully capture the features and relative complexity of each type of user.

### ML-based evaluation metrics

The fourth and last comparative study uses ML classification models to assess the similarity of features among the two datasets. More specifically, we used train on synthetic, test on real (TSTR), and train on real, test on real (TRTR)^59^. TSTR evaluates the performance of the synthetic data by training a model (classifier) with synthetic data and testing it on real data. This way, a synthetic dataset has high quality only if the classifier trained with synthetic data performs close to the classifier trained with real data (TRTR). We applied a 1D convolutional neural network (CNN) to classify five types of prosumers, i.e., with the hyperparameters reported in Table 2.

**Table 2:** Hyperparameters for 1D-CNN.

Hyperparameter	Value
Optimizer	Adam
Batch size	24
Number of hidden layers	4
Cov1D	
Kernel size	3
Filters	128
Dropout	
Rate	0.2
MaxPooling1D	
Pool size	2
Flatten	—
Relu	
Units	64

**Table 3:** Weighted Permutation Entropy for Real data and Synthetic data.

User types	Import (real)	Import (synthetic)	Export (real)	Export (synthetic)
PV workday	0.82	0.94	0.46	0.72
PV other day	0.84	0.96	0.50	0.73
PV&ESS workday	0.82	0.94	0.50	0.75
PV&ESS other day	0.84	0.95	0.50	0.78
EV&PV&ESS workday	0.80	0.93	0.54	0.70
EV&PV&ESS other day	0.81	0.96	0.54	0.83
EV&PV workday	0.83	0.93	0.53	0.74
EV&PV other day	0.84	0.96	0.56	0.74
EV workday	0.90	0.98	—	—
EV other day	0.91	0.99	—	—

**Fig. 9:**
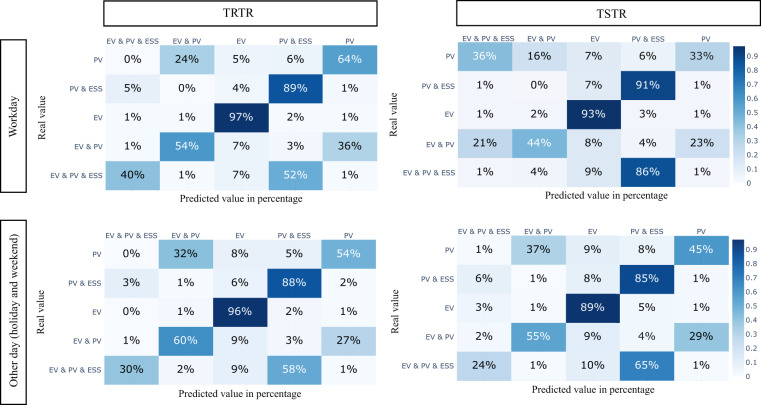
Confusion matrices for users.

Applying the same classifier, we tried to determine the prosumer’s types in the workday and other days’ datasets. The results of the four combinations are presented as confusion matrices in Fig. 9. For most user types, the classifier shows similar results on TRTR and TSTR, which proves the existence of similar features in both real and synthetic datasets. Comparing TSTR with TRTR in Fig. 9, we find the general numerical relationship for the predicted results and ground truth are highly similar between real data and synthetic data. The overall accuracy, precision, sensitivity (recall) and specificity are also provided in Tables 4, 5. We find a 10% gap in accuracy between synthetic and real datasets, which is acceptable for a synthetic dataset, e.g., see Table 6 in^60^. For workdays’ classification in Fig. 9, PV users could be wrongly identified as EV & PV & ESS compared to TSTR. One potential reason could be the similar complexity values of the two user types in the synthetic dataset compared to the real dataset, as reported in Table 3, indicating their frequencies and amplitudes on fluctuations are similar.

**Fig. 10:**
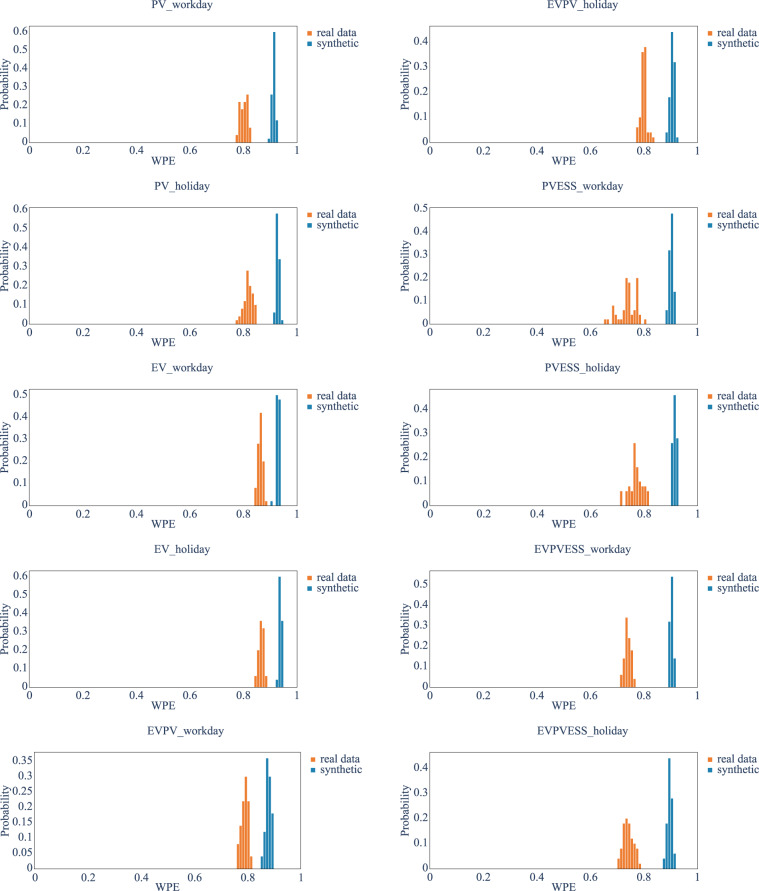
WPE for different types of users (50) in the yearly manner (blue: synthetic data, orange: real data).

**Table 4:** Classification performance comparison for workday data.

Data types	Accuracy	Precision	Recall (Sensitivity)	Specificity
Workday TRTR	66%	0.72	0.67	0.97
Workday TSTR	55%	0.57	0.54	0.95

**Table 5:** Classification performance comparison for other day data.

Data types	Accuracy	Precision	Recall (Sensitivity)	Specificity
Other day TRTR	66%	0.70	0.63	0.97
Other day TSTR	61%	0.62	0.61	0.96

## Usage Notes

### Limitations

The first limitation of our synthetic dataset is the hourly resolution, which is insufficient for some applications, such as energy disaggregation and power quality analysis. Also, research shows using hourly data for PV users’ self-consumption estimation can yield up to a 9% over-estimation due to the information loss^61^. However, the presented synthetic dataset can be used for many studies, e.g., demand response, reverse power flow from prosumers, examining the impact of different adoption rates, and demand-side management. Another limitation is the complexity of synthetic data tends to be overestimated due to the structure of CTGAN, as we discussed in the Data Validation section. Last but not least, the dataset does not fully take into account the prosumers’ behavioural habits and changes at the appliance level over time since the seed dataset does not have labels for each end-user’s appliances. One potential improvement to include additional behavioural stochasticity associated with appliances’ electricity demand is using a bottom-up physics-based model, e.g., StROBe library, when the users want to add certain appliances with knowledge on the distributions of detailed physical parameters^12^. However, this will produce a synthetic dataset with higher complexity beyond the results reported in the ‘Information theory metrics’ section, which is not desirable.

## Data Availability

The real data used as the input of CTGAN is unavailable due to regulations around consumers’ privacy^18^. Others wishing to repeat the work or perform studies with the raw data should approach Watts A/S^13^. The code for data validation and analysis is available in the public repository of Figshare^51^.
